# Clinician Delivery Strategies for Exercise-Based Pain Management in Dementia: A Qualitative Study

**DOI:** 10.1093/geroni/igaf122.3732

**Published:** 2025-12-31

**Authors:** Annalisa Na, Amy Kwok, Ben Senderling, Jenna Lisa, Peter Gliebus, Julie Fritz, Joke Bradt, Laura Gitlin

**Affiliations:** Drexel University, Philadelphia, Pennsylvania, United States; Drexel University, Philadelphia, Pennsylvania, United States; Drexel University, Philadelphia, Pennsylvania, United States; Drexel University, Philadelphia, Pennsylvania, United States; Baptist Health, Boca Rotan, Florida, United States; University of Utah, Salt Lake City, Utah, United States; Drexel University, Philadelphia, Pennsylvania, United States; Drexel University, Philadelphia, Pennsylvania, United States

## Abstract

Chronic pain affects over 50% of community-dwelling people living with dementia (PLWD), compromising function and quality of life. Despite its prevalence and poor outcomes, evidence-based, non-pharmacological interventions for PLWD remain limited. Guided exercise programs at therapeutic doses targeting underlying pain mechanisms (e.g., osteoarthritis) and associated impairments (e.g., range of motion, weakness) show promise but lack applicability to PLWD. Hence, clinicians rely on trial-and-error, resulting in care variability, resource waste, diminished care quality, and highlighting the need for intervention development. An initial step in developing and testing exercise-based pain interventions is to assess the range of current practices used by clinicians. This qualitative study explores strategies currently used by clinicians to engage PLWD in exercise-based pain management. Sixteen clinicians with at least two years of experience managing pain and working with PLWD in non-institutional settings (e.g., outpatient, home-health) participated in focus groups. We analyzed deidentified transcripts using conventional content analysis and identified an overarching theme that clinicians adopted person-centered approaches. Clinicians explained person-centered approaches include 1) Being flexible with aiding and cueing based on real-time assessments; 2) Employing hands-on strategies to direct attention, support movement recognition, and enhance body awareness. (3) Allowing personalized selection of music through accessible technology to offer rhythmic cueing, foster motivation, support sensory regulation, and create supportive environments. These clinician-described strategies demonstrate that one-size-fits-all models are inadequate and emphasize the need for adaptive, person-centered approaches. These findings support the development of an evidence-informed framework for exercise-based pain management tailored to community-dwelling PLWD.

